# Evaluation of open bite closure using clear aligners: a retrospective study

**DOI:** 10.1186/s40510-020-00325-5

**Published:** 2020-08-24

**Authors:** Kayla Harris, Kenji Ojima, Chisato Dan, Madhur Upadhyay, Abdulrahman Alshehri, Chia-Ling Kuo, Jinjian Mu, Flavio Uribe, Ravindra Nanda

**Affiliations:** 1 Division of Orthodontics, Department of Craniofacial Sciences, University of Connecticut School of Dental Medicine, Farmington, CT USA; 2 Tokyo, Japan; 3 Division of Orthodontics, Department of Preventive Dental Sciences, College of Dentistry, Jazan University, Jazan, Saudi Arabia; 4 CICATS/Department of Community Medicine/Institute for System Genomics, University of Connecticut Health, Farmington, CT USA

**Keywords:** Clear aligners, Open bite, Extrusion, Intrusion, Autorotation

## Abstract

**Objectives:**

To evaluate the dental and skeletal effects that occur in the correction of anterior open bite with clear aligners.

**Materials and method:**

In this single-center retrospective study, the mechanism of anterior open bite closure using clear aligners (Invisalign, Align Technology, Santa Clara, CA, USA) was evaluated by cephalometric superimposition based on records of patients consecutively treated by a single, experienced Invisalign provider. Inclusion criteria consisted of anterior open bite (overbite < 0.5 mm), adult patients (18+) at the beginning of treatment, consecutive records, and good quality pre- and post-treatment records, where the required landmarks were clearly visible.

**Results:**

A total of 45 patients were included for data analysis with a mean age of 30.73 ± 8.0 years and initial open bite of − 1.21 ± 1.15 mm. During treatment, the upper incisors showed significant (*p* < 0.05) retraction [U1-SN′(°) = − 10.91 ± 6.95°], [U1-SN′_perp_(mm) = − 2.57 ± 1.75 mm] and extrusion [U1-SN′(mm) = 1.45 ± 0.89 mm]. The lower incisors also showed significant retraction [IMPA(°) = − 3.73 ± 4.91°), (ΔL1-MP′_perp_ (mm) = − 1.08 ± 1.59] and extrusion (ΔL1-MP′(mm) = 0.53 ± 0.74). Regarding molar position, no significant changes were noted in the anteroposterior position of the upper [ΔU6-SN′_perp_(mm) = 0.01 ± 1.08 mm] and lower molar [ΔL6-MP′_perp_(mm) = 0.03 ± 0.87 mm]; however, there was a statistically significant intrusion of the upper [ΔU6-SN′(mm) = − 0.47 ± 0.59 mm] and lower molar [ΔL6-MP′(mm) = − 0.39 ± 0.76 mm].

**Conclusion:**

Open bite closure with clear aligners occurred due to a combination of maxillary and mandibular incisor extrusion and maxillary and mandibular molar intrusion, with slight mandibular auto rotation. Significant retraction of maxillary and mandibular incisors was also observed with treatment. Clear aligners are effective in reducing/controlling the vertical dimension in open bite patients.

## Introduction

Advances is clear aligner technology have expanded the scope of clear aligners from treatment of simple malocclusions to more complex approaches such as treatment of anterior open bites [1]. Aligners may be advantageous in treating this type of malocclusion as they do not produce the same extrusive effect on the posterior teeth as would occur with traditional brackets. Straight wire mechanics tend to extrude the posterior teeth which tend to worsen the anterior open bite. Conversely, anecdotal evidence shows that aligners may help intrude posterior teeth because of the thick plastic covering the posterior teeth and the patient’s natural masticatory forces [2, 3]. It has also been theorized that aligners can help with habit modification such as tongue thrusting due to the presence of plastic covering the anterior teeth. Although these statements are not evidence based, there have been case reports showing successful treatment of patients with moderate to severe anterior open bites [1, 4, 5].

A retrospective study evaluating the ability of clear aligners to control the vertical dimension in deep and open bite patients reported that the primary mechanism of open bite correction occurred by incisor extrusion with a median value of 1.5 mm. In this study, the sample size for the open bite group was small, therefore no strong conclusions could be made regarding treatment of open bites [6]. In contrast, another retrospective study evaluating 30 adult open bite patients using cephalometric analysis found that the correction of the open bite was primarily occurring by counterclockwise rotation of the mandible resulting from lower molar intrusion [7]. Recently, a study compared clear aligners for open bite correction to a group with fixed appliances in patients with a hyperdivergent growth pattern. The fixed appliance group included patients treated with extractions and TADs (Temporary Anchorage Devices), while the clear aligner group did not. The results did not show any significant differences in the treatment outcomes between the groups, suggesting that clear aligners might have the same efficacy at controlling the vertical dimension as fixed appliances with additional auxiliaries, such as TADs in hyperdivergent patients [8]. Based on this limited evidence, additional research is needed to better clarify the mechanism by which aligners are primarily used in open bite correction.

The limited evidence-based research shows conflicting results with a low sample size. Hence, the mechanism of open bite correction is not clear. There is a clear need to understand which specific tooth movements are contributing toward open bite correction. Therefore, the primary objective of this study is to quantify dental and skeletal changes associated with open bite closure using clear aligners. Our null hypothesis is that there is no difference in the pre- and post-treatment molar position in the vertical dimension with clear aligner therapy.

## Materials and methods

This study was a single-center retrospective evaluation of the mechanism of anterior open bite closure using clear aligners (Invisalign, Align Technology, Santa Clara, CA, USA) obtained from the records of adult patients consecutively treated by a single, experienced Invisalign provider. It was approved by the Institutional Review Board at the University of Connecticut Health (IRB#18X-138-1). Patient records were screened at a private practice in Tokyo, Japan. The consecutive records were screened from all the records in the office, with no influence of the practitioner that delivered the care. The records were de-identified and reviewed at the Division of Orthodontics, University of Connecticut Health, from October 2018 to April 2019 by one primary investigator (K.H.) and two secondary investigators (A.A. and M.U.).

The power analysis was performed using the computer application G power 3.1. The minimal clinically important difference (MCID) of 0.5 mm ± 1.0 mm in the vertical position of first molar was considered clinically significant based on the results by Khosravi et al. [6]. The initial calculation for sample size estimation indicated that for a power of 80% and an alpha error of 0.05 (i.e., *p* < 0.05 was deemed as statistically significant), we needed 34 patients. However, the actual sample size of 45 patients gave us 91% power to detect a change in the molar position at the 5% significance level.

All records of consecutively treated patients with anterior open bite treated between 2007 and 2018 were included for review. A total of 250 patient records were initially screened. Forty-five patients were ultimately selected for evaluation based on the following inclusion criteria: anterior open bite (overbite < 0.5 mm), age 18+ at the beginning of treatment, consecutive records, and good quality pre- and post-treatment records, where the required landmarks were clearly visible. We excluded any patients with significant medical history (syndromes, etc.), treatment involving orthodontic appliances other than Invisalign, or a treatment plan involving extraction of premolars, significant antero-posterior (AP) molar correction, surgery, or the use of skeletal anchorage devices.

Data obtained from patient records included information from the Invisalign Treatment Overview and radiographs, specifically lateral cephalograms and panoramic films, from two time points: pre-treatment (T0) and post-treatment (T1). The post-treatment timepoint was taken after all refinements were complete. All data was collected and de-identified by a single examiner K.H. Each patient was assigned a unique number which was used to correlate the information in the Data Collection Form with the radiographs. To differentiate the pre-treatment vs. post-treatment radiographs, a letter code was assigned where the T0 lateral cephalogram was indicated by the letter “A” and the T1 lateral cephalogram was indicated by the letter “B”. The Data Collection Form and any radiographs collected were kept on a password-protected computer and any hard copies were kept in a locked cabinet. Patient demographic information such as age, gender, and ethnicity were also collected. Information collected from the Invisalign Treatment Overview included duration of treatment, number of aligners, frequency of aligner changes, number of refinements, and ClinCheck characteristics such as use of auxiliaries (i.e., elastics), attachments, inter-proximal reduction, and planned posterior intrusion.

**Fig. 1.**
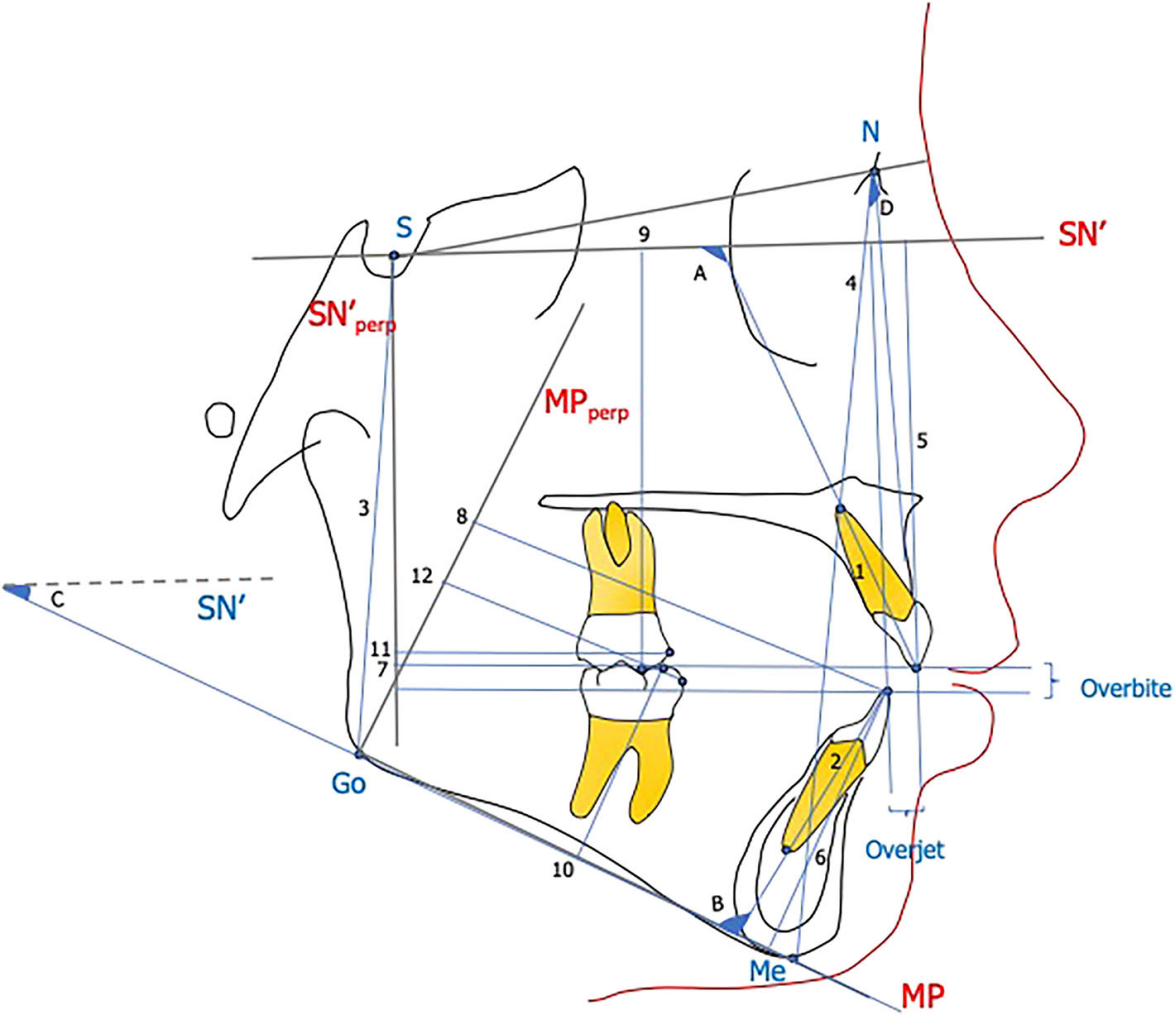
*Angular measurements (°).* A, U1-SN′: angle between SN′ and long axis of upper incisor; B, IMPA: angle between Go-Me and long axis of lower incisor; C, SN′-MP: angle between SN′ and MP (Go-Me); D, ANB: angle between N-A and N-B. *Linear measurements (mm)*. 1, UI length: distance from upper incisor tip to apex; 2, LI length: distance from lower incisor tip to apex; 3, PFH: shortest distance from S-Go; 4, AFH: shortest distance from N-Me; 5, U1-SN′: distance from tip of upper incisor to SN′; 6, L1-MP: distance from lower incisor tip to MP; 7, U1-SN′_perp_: distance from tip of upper incisor to SN′_perp_; 8, L1-MP_perp_: distance from lower incisor tip to MP perp; 9, U6-SN′: distance from upper molar MB cusp to SN′; 10, L6-MP: distance from lower molar MB cusp to MP_;_ 11, U6-SN′_perp_: distance from tip of upper molar MB cusp to SN′_perp_; 12, L6-MP_perp_: distance from lower molar MB cusp to MP_perp_; overbite: distance from tip of U1 to tip of L1 perpendicular to SN′_perp_; overjet: distance from tip of U1 to tip of L1 perpendicular to SN′

Cephalometric tracing and landmark identification were performed on acetate tracing paper by a single examiner. A total of 14 landmarks were identified on the pre-treatment and post-treatment lateral cephalogram (Fig. 1). The landmarks for the pre-treatment and post-treatment radiographs were traced sequentially in order to reduce landmark identification error. Horizontal and vertical reference planes were traced on the T0 lateral cephalogram and transferred to the T1 image using the structural superimposition method [9, 10]. For the cranial base superimposition, the horizontal reference plane was defined as Sella (S)-Nasion (N) minus 7° (SN′) and the vertical reference plane was a line perpendicular (perp) to SN′ and passing through sella (SN′_perp_) [10]. For the mandibular superimposition, the mandibular plane (MP) served as the horizontal reference plane and was defined by the lower border of the mandible passing from gonion to menton (Go-Me). A line perpendicular to the mandibular plane passing through gonion (MP_perp_) served as the vertical reference plane. A total of 18 measurements were performed (4 angular and 14 linear) based on the horizontal and vertical reference planes on the pre and post treatment cephalometric radiographs (Fig. 1).

Forty radiographs were randomly selected, re-traced, and re-measured after a period of 4 weeks by the same examiner (K.H.) as well as a second examiner (A.A.) to determine inter- and intra-rater reliability. The lateral cephalometric images used in the study were taken on two different cephalometric machines. To account for any magnification errors between the patients, a magnification factor was determined for the second machine and applied to the images taken on that machine. This was done in consultation with a board certified oral maxillofacial radiologist.

### Statistical analysis

Pre-treatment and post-treatment measurements and the differences between the two were summarized by their respective means and standard deviations. Pre- and post-treatment differences were tested against no differences by paired *t* tests and were compared among mild, moderate, and severe baseline severity groups, using linear regression models. The least square means and 95% confidence intervals were reported. Post-hoc pairwise comparisons were conducted using Tukey’s method if the overall test for any between-group difference was statistically significant. Between-rater and within-rater agreement on *z*-transformed (standardized) pre- and post-measurements were assessed by intra-class correlations. A *p* value smaller than 0.05 was deemed to be statistically significant. All the statistical analyses were performed in R 3.5.3 (The R Project for Statistical Computing; https://www.r-project.org/).

## Results

An initial screening of 250 consecutively treated adult patients with anterior open bite was performed. Based on the inclusion and exclusion criteria, 58 patients were deidentified and included for further review. Thirteen patients were excluded for various reasons: incomplete records (*n* = 8), vertical overlap of incisors on the pre-treatment cephalometric analysis (*n* = 2), extraction of second molars (*n* = 1) and magnification errors (*n* = 2). A total of 45 patients were included for data analysis. The mean age of patients at the beginning of treatment was 30.73 ± 8.0 years (range 18–53). All were classified at Cervical Vertebral Maturation Stage V (CVMS V) or greater based on the T0 lateral cephalogram [11]. Most of the patients included were female (*n* = 41; 91%) while the remaining were male (*n* = 4; 9%). The ethnicity of the patient population was primarily Japanese (*n* = 44; 98%), with only 1 patient of Chinese origin (*n* = 1; 2%). The mean treatment duration was 14.04 ± 6.25 months (range 6.5–34) with an average use of 73.89 ± 36.37 aligners (Table 1). Aligners were changed every 5 days. Acceledent devices were used for 20 min/day by all, except 2 patients that were treated prior to the availability of this device in 2009.

**Table 1:**
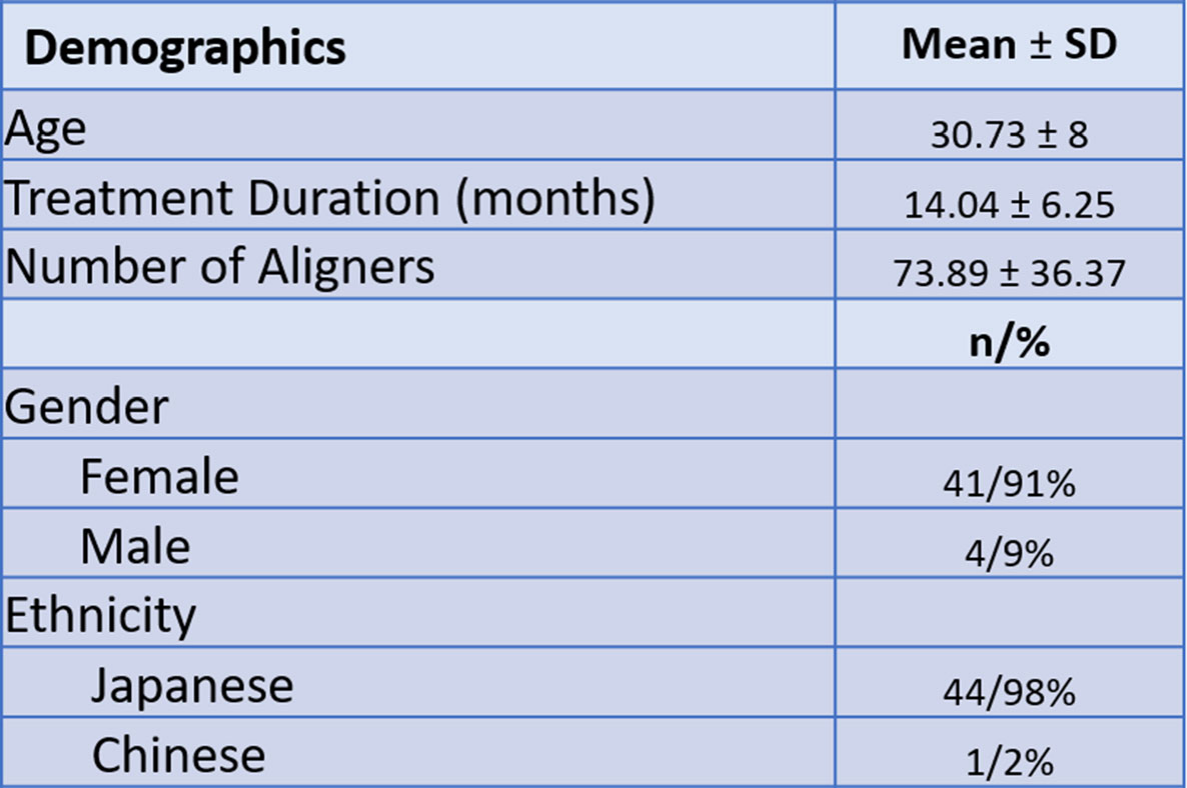
Patient demographics

The study population was stratified into three groups based on the initial severity of open bite (mean 1.21 ± 1.15 mm): mild < 0 to – 1 mm (*n* = 20; 44.4%), moderate < –1.1 mm to – 2 mm (*n* = 16; 35.6%), and severe: < – 2.1 mm (*n* = 9; 20%). Classification was also carried out to determine the skeletal open bite severity based on the initial mandibular plane angle (SN′-MP°). Three groups were identified: low angle < 30° (*n* = 14; 31.2%), normal angle 31–36° (*n* = 20; 44.4%), and high angle > 37° (*n* = 11; 24.4%).

**Fig. 2:**
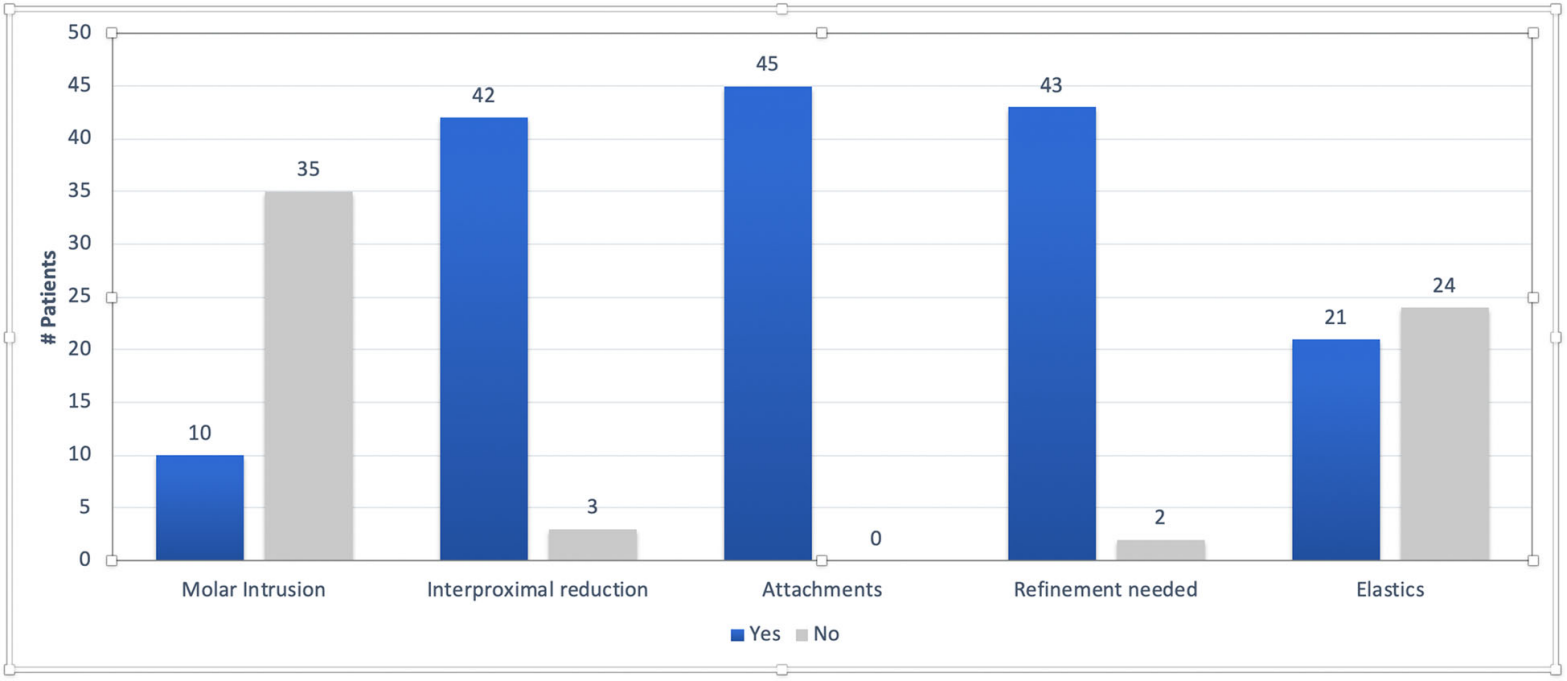
ClinCheck characteristics for the full treatment T0–T1 (*n* = 45)

The treatment plans (ClinCheck® software, Invisalign©) for the included patients included various strategies for open bite closure including molar intrusion, use of inter-proximal reduction (IPR), attachments (Invisalign® attachments) for incisor extrusion, and refinement trays if required (Fig. 2). It is important to note that only 17 patients were specifically planned for molar intrusion in the “ClinCheck” while the remaining had no prescribed mechanism of maintaining the posterior vertical dimension. This may be because some patients in this study were treated prior to 2011 when the algorithms for posterior intrusion were introduced in 2011 with Invisalign’s G4 protocol. All patients (*n* = 45) had attachments in some form to help with open bite closure and 42 patients (93.3%) had IPR prescribed in their treatment plan. Only 3 patients did not require any refinement. The number of refinements ranged from 0 to 3 for the sample with a mode of 1.

Intra-class correlation coefficients (ICC) were determined for both inter-rater and intra-rater reliability (Table 2). For intra-rater reliability, the ICC ranged from 0.84 to 0.98. For inter-rater reliability, the ICC ranged from 0.83 to 0.99, showing good to excellent measurement reliability.

**Table 2:** Intra-rater and inter-rater reliability

	ICC-intra-rater 95% CI (lower bound, upper bound)	ICC-inter-rater 95% CI (lower bound, upper bound)
U1-SN′ T0(°)	0.94 (0.85, 0.97)	0.95 (0.87, 0.98)
U1-SN′ T1 (°)	0.89 (0.75, 0.96)	0.88 (0.71, 0.95)
U1-SN′_perp_ T0 (mm)	0.88 (0.73, 0.95)	0.95 (0.87, 0.98)
U1-SN′_perp_ T1 (mm)	0.85 (0.66, 0.94)	0.92 (0.81, 0.97)
U1-SN′ T0 (mm)	0.86 (0.69, 0.94)	0.85 (0.65, 0.94)
U1-SN′ T1 (mm)	0.84 (0.69, 0.93)	0.83 (0.61, 0.93)
U6-SN′ T0 (mm)	0.86 (0.67, 0.94)	0.92 (0.81, 0.97)
U6-SN′ T1(mm)	0.86 (0.69, 0.94)	0.92 (0.81, 0.97)
IMPA T0(°)	0.95 (0.88, 0.98)	0.98 (0.96, 0.99)
IMPA T1(°)	0.91 (0.78, 0.96)	0.98 (0.95, 0.99)
L1-MP_perp_ T0 (mm)	0.85 (0.67, 0.94)	0.97 (0.92, 0.99)
L1-MP_perp_ T1 (mm)	0.86 (0.68, 0.94)	0.96 (0.91, 0.99)
L1-MP T0 (mm)	0.98 (0.94, 0.99)	0.98 (0.95, 0.99)
L1-MP T1 (mm)	0.98 (0.95, 0.99)	0.99 (0.97, 1.00)
L6-MP T0 (mm)	0.97 (0.92, 0.99)	0.98 (0.94, 0.99)
L6-MP T1 (mm)	0.97 (0.93, 0.99)	0.99 (0.96–0.99)

*ICC* intra-class correlation coefficient; *CI* confidence interval

Significant changes (*p* < 0.05) were noted for all parameters except ANB (°), U6-SN′_perp_ (mm), and L6-MP_perp_ (mm) (Table 3). There was a statistically significant decrease in anterior facial height (AFH) seen during treatment [AFH(mm) = – 1.17 ± 1.46; *p* = 0.000] (Table 3).

**Table 3:**
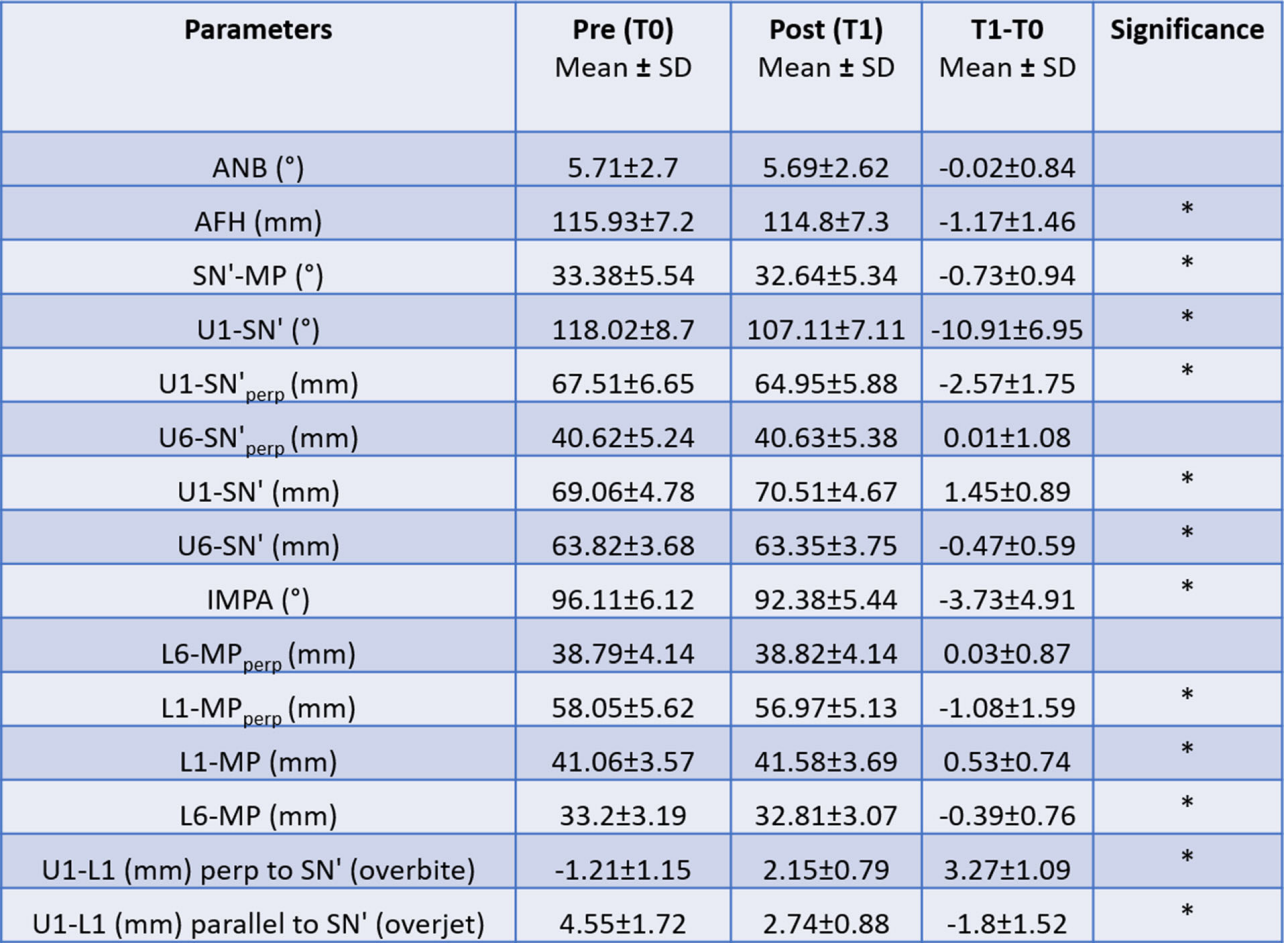
Mean pre-treatment (T0), post-treatment (T1), and treatment difference (T1–T0) values

*p < 0.05

The maxillary and mandibular incisors showed significant retraction and extrusion. (Table 3). Regarding molar position, no significant changes were noted in the anteroposterior position of the upper and lower molar (Table 3). However, there was a statistically significant intrusion of the upper and lower molar. As expected, this was accompanied by a counterclockwise rotation of the mandible. A significant association was found between the autorotation of the mandible and the total amount of molar intrusion. No statistically significant association was found between the patients planned for molar intrusion and patients who showed it (Table 4). This indicates that molar intrusion is occurring irrespective of the initial treatment plan.

**Table 4:** Mean molar intrusion related to molar intrusion planned/not planned in ClinCheck

	Planned molar intrusion (yes/no)		*p* value
	Yes	No	
U6-SN′ T2–T1 (mm)	− 0.62 ± 0.72	− 0.43 ± 0.56	0.44
L6-MP T2–T1 (mm)	− 0.38 ± 0.78	− 0.39 ± 0.76	0.95
Molar intrusion sum	− 1 ± 1.22	− 0.82 ± 0.95	0.68

*p < 0.05

Linear regression analysis was completed to assess the difference in the outcomes depending on the initial severity of open bite (Table 5). There was a significant difference between the mild, moderate, and severe open bite groups for ANB (°), SN-MP (°), L6-MP (mm), and U1-L1 (mm) perp to SN′ (overbite) measurements. A significant difference in the extent of lower molar intrusion [L6-MP (mm)] was present between the mild open bite and severe open bite groups. Linear regression conducted to assess the difference in the outcomes regarding the initial SN′-MP angle revealed no significant differences in any of the parameters between the low-, normal-, and high-angle groups (Table 6).

**Table 5:**
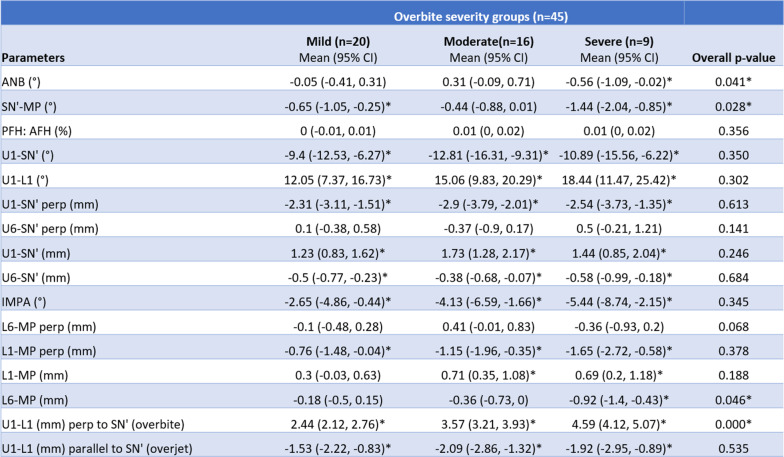
Mean treatment changes (T1–T0) based on pre-treatment overbite severity

*p < 0.05

**Table 6:**
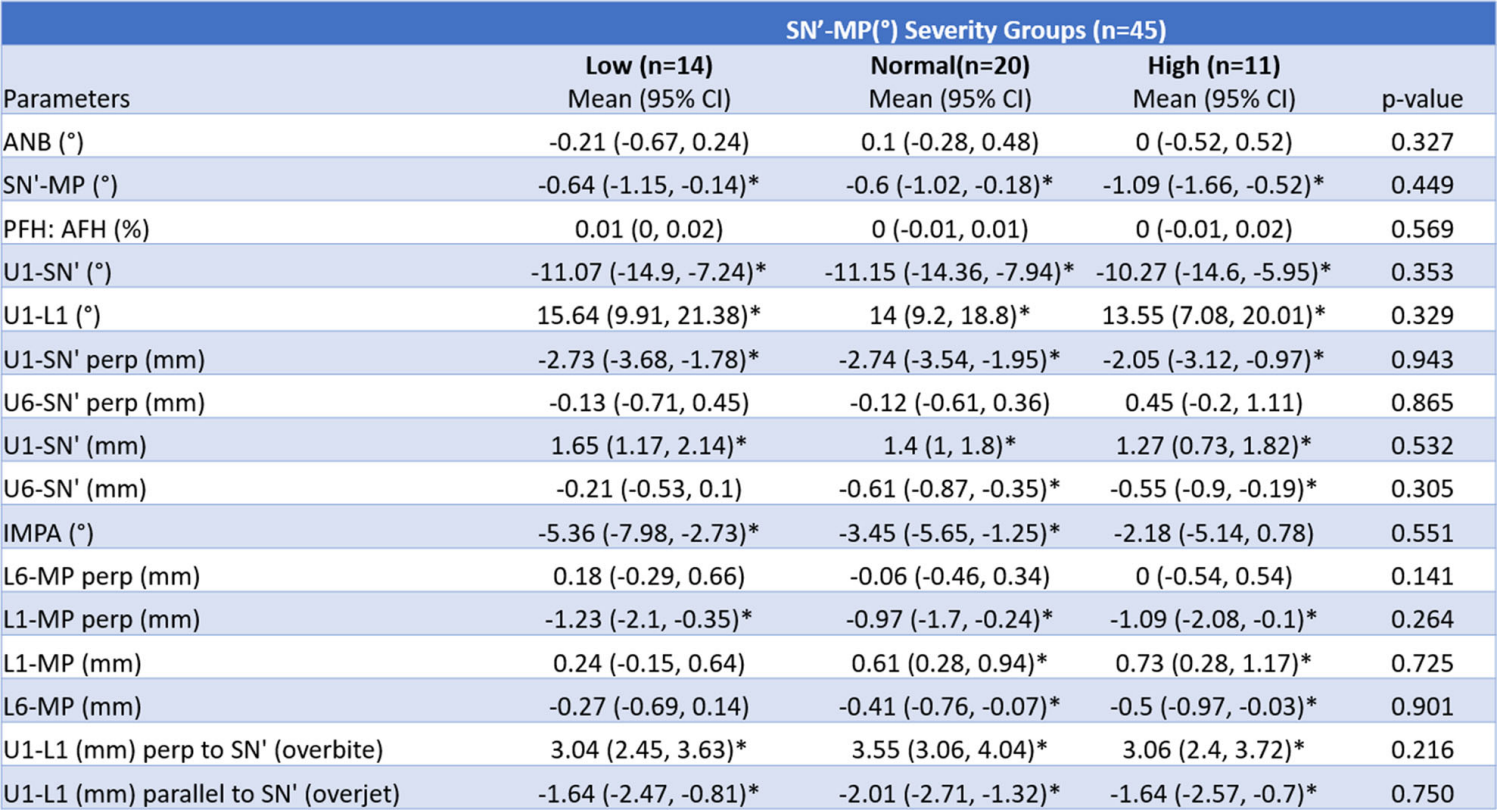
Mean treatment changes (T1–T0) based on pre-treatment mandibular plane angle severity

*p < 0.05

## Discussion

The aim of this study was to analyze the dental and skeletal effects resulting from anterior open bite correction with clear aligners. The average initial open bite for our patients at T0 was – 1.21 ± 1.15 mm. This pre-treatment severity was similar to the study by Khosravi et al. [6] which had an initial open bite of – 1.1 mm, but less than those described by Moshiri et al. [7] and Garnett et al. [8]. Our results show that the average overbite change during treatment was 3.27 ± 1.09 mm. While our initial pre-treatment open bite severity was less than previous studies, the overall overbite change is greater than most and like that reported by Moshiri et al. [7] who found an overbite change of 3.4 ± 1.4 mm. In general, there were more dental changes than skeletal changes contributing to the overbite closure in this study. Both the upper and lower incisors showed extrusion while the molars were intruded. Therefore, the null hypothesis was rejected and the alternate hypothesis was accepted.

Fixed appliances tend to extrude the posterior teeth during treatment which can cause worsening of an anterior open bite, especially in non-growing individuals [12, 13]. As a result, a greater extrusion of the anterior teeth may become necessary to obtain positive overbite. This study showed significant amounts of maxillary and mandibular molar intrusion followed by counterclockwise rotation of the mandible. The aligners were not only successful in maintaining the vertical dimension of the open bite patients but were also able to reduce it, albeit by only small amounts. This “bite block effect” has been previously described with aligners and has been attributed to the thickness of material covering the posterior teeth coupled with the biting force exerted by the patients [2]. Our findings also agree with a recent study evaluating the effects on the maxillary molars during first premolar extraction with Invisalign. The authors found intrusion of the maxillary mesiobuccal cusps of 0.6 mm which is almost the same amount that we found in our study (0.47 mm) at the same location [14].The intrusive effect created on the posterior dentition was however not intentional. No correlation was found between the measured molar intrusion and planned molar intrusion. This may indicate that the bite deepening effect of the aligners is occurring in all patients unless measures are taken to prevent it, such as anterior bite ramps for deep bite cases [6]. This finding contradicts what was reported by Moshiri et al. [7]. In their study, they reported that the intrusion of the posterior teeth with aligners must be programmed into the trays; however, this statement was not supported by evidence. A bite block effect is generally associated with posterior blocks of at least 3–4 mm thickness as they are thick enough to exceed the patient’s freeway space and create a sustained intrusive effect on the posterior teeth [3, 15–17]. However, aligners may not have substantial thickness to create such an effect.

The overall aim of this study was to evaluate the dental and skeletal effects of open bite closure with aligners and to determine the mechanism of open bite closure. Due to the retrospective nature of this study, there were several limitations including lack of a control group and the inability to control all treatment variables. Although a general treatment protocol from the treating clinician could be described, each case was treatment planned appropriately based on the individual malocclusion. Therefore, the features prescribed in each patients’ ClinCheck varied. Also, during treatment the time frame in which the patients were included in this retrospective study, significant improvements to the treatment protocols have been introduced to the Invisalign system. Nonetheless, this study provides some insight on the mechanisms that are primarily responsible for anterior open bite correction. Above all, there is a need for randomized controlled trials which can prospectively follow patients undergoing anterior open bite correction with clear aligners and compare those results with a similarly matched groups treated with fixed appliances.

## Conclusions

1. Open bite closure with clear aligners occurred due to a combination of maxillary and mandibular incisor extrusion and maxillary and mandibular molar intrusion, leading to mandibular auto rotation and reduction in anterior facial height. Therefore, our null hypothesis has been rejected.
2. Maxillary and mandibular incisors were also significantly retracted during treatment.
3. Clear aligners are effective in reducing/controlling the vertical dimension in open bite patients.

## Data Availability

The datasets used and/or analyzed during the current study are available from the corresponding author on reasonable request.

## Abbreviations

TADs: Temporary Anchorage Devices
MCID: Minimal Clinically Important Difference
AP: Antero-posterior
T0: Pre-treatment
T1: Post-treatment
S: Sella
N: Nasion
Perp: Perpendicular
MP: Mandibular plane
Go: Gonion
Me: Menton
U6: Maxillary first molar
L6: Mandibular first molar
U1: Maxillary central incisor
L1: Mandibular central incisor
AFH: Anterior facial height
ICC: Intra-class correlation coefficient
IPR: Inter-proximal reduction
CVMS: Cervical vertebral maturation stage
